# A Case of Pathological Complete Response and Resolution of Dermatomyositis Following Neoadjuvant Chemotherapy in HER2-Positive Early Breast Cancer

**DOI:** 10.3390/curroncol28030182

**Published:** 2021-05-24

**Authors:** Marta Piras, Martina Panebianco, Matteo Garibaldi, Michela Roberto, Gioia Merlonghi, Patrizia Pellegrini, Paolo Marchetti

**Affiliations:** 1 Oncology Unit, Department of Clinical and Molecular Medicine, Sant’Andrea Hospital, University “La Sapienza”, 00189 Rome, Italy; marta.piras@uniroma1.it (M.P.); martina.panebianco@uniroma1.it (M.P.); patrizia.pellegrini@uniroma1.it (P.P.); paolo.marchetti@uniroma1.it (P.M.); 2 Neuromuscular and Rare Disease Centre, Department of Neuroscience, Mental Health and Sensory Organs (NESMOS), SAPIENZA University of Rome, Sant’Andrea Hospital, 00189 Rome, Italy; matteo.garibaldi@uniroma1.it (M.G.); gioia.merlonghi@uniroma1.it (G.M.)

**Keywords:** dermatomyositis, paraneoplastic, breast cancer, neoadjuvant chemotherapy, trastuzumab

## Abstract

Introduction. Dermatomyositis (DM) is an idiopathic inflammatory myopathy (IIM) mainly characterized by subacute muscle weakness and skin rash sometimes associated with malignancy. Case Presentation. A 61-year-old female was admitted to our hospital because of progressive proximal muscular weakness, heliotropic rash and left breast rash. Muscle biopsy findings were consistent with dermatomyositis (DM). A full panel of myositis associated (MAA) and specific antibodies (MSA) revealed the presence of anti-nuclear antibodies (1:160, speckled), Anti-Ro52 and anti TIF1-γ antibodies. A whole body Computed Tomography Scan showed three left mammary nodules and homolateral axillary lymphadenopathy. The breast biopsy confirmed the diagnosis of ductal carcinoma. Patient was initiated to neoadjuvant chemotherapy followed by surgery for cancer, and corticosteroid and intravenous immunoglobulins for DM with a complete resolution of muscle weakness and pathological complete response of breast cancer. *Discussion and conclusion.* Similar cases in literature are commonly referred to a first-line surgery and the role of neoadjuvant chemotherapy is debatable.

## 1. Case Presentation

A 61-year-old woman presented to our hospital with a 4-month history of a heliotropic rash, proximal muscle weakness, dysphagia and a mass of external left breast quadrants.

**Figure 1:**
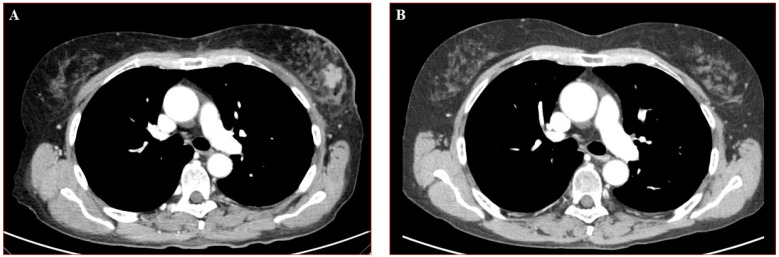
Radiological complete response documented at pre (**A**) and post (**B**) neoadjuvant treatment images.

Laboratory findings showed creatine kinase (CK) elevation (1024 IU/L). Electromyography (EMG) showed myopathic findings on proximal muscles of upper limbs with fibrillation potentials and positive sharp waves. Deltoid muscle biopsy showed fiber size variability with perifascicular atrophy, muscle fiber necrosis and regeneration, perimysial inflammatory infiltrates and sarcolemmal/cytoplasmic expression of Major Histocompatibility Complex class I (MHC-I), consistent with dermatomyositis (shown in Figure 1). A full panel of myositis associated (MAA) and specific antibodies (MSA) revealed the presence of anti-nuclear antibodies (1:160, speckled), Anti-Ro52 (SSA) and anti TIF1-γ antibodies.

With the aim of assessing the breast lesion, the patient was subjected to mammary ultrasound and mammography, which detected on the left, at the level of the external mammary quadrants, a solid hypoechogenic and vascularized lesion, irregularly polylobate, with some microcalcifications, at least 45 mm in size. In the homolateral axillary cavity some suspected lymphadenopathies were concurrent, the largest was 15 mm.

Whole body Computed Tomography (CT) revealed the presence of three left breast mammary nodules (maximum diameter (MD) 36 mm), and cutaneous thickening in homolateral pectoral seat and left axillary lymph nodes (MD 11 mm), in the absence of metastasis (T2N2M0 clinical stage). The breast biopsy specimen from left mammary nodule confirmed infiltrating ductal carcinoma, grade 2, Estrogen Receptor (ER)-positive (30%), Progesterone Receptor (PR)-negative, HER2-positive (2+ by IHC and FISH positive) and Ki67 22%. The pathological nature of lymphadenopathies was confirmed by fine-needle aspiration.

From September 2019 to March 2020, the patient received neoadjuvant chemotherapy with cyclophosphamide (600 mg/m^2^) and doxorubicin (60 mg/m^2^) every two weeks for four cycles followed by weekly paclitaxel (80 mg/m^2^) and trastuzumab (4 mg/kg loading dose, followed by 2 mg/kg) for 12 administrations.

During the neoadjuvant treatment, the patient was treated with intravenous immunoglobulins (IVIGs) and oral prednisone as maintenance dosage of 25 mg/die for dermatomyositis with clinical improvement.

**Figure 2:**
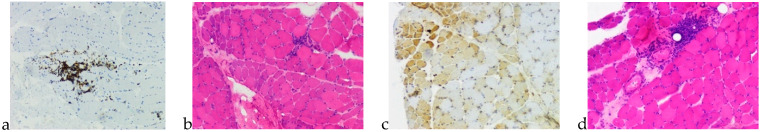
Deltoid muscle biopsy showed (**b**) fiber size variability with perifascicular atrophy, fiber necrosis and regeneration, (**a**,**d**) perimysial inflammatory infiltrates and (**c**) sarcolemmal expression of MxA (Myxovirus Resistance Protein).

At the end of neoadjuvant treatment for breast cancer, she had a major clinical improvement of dermatomyositis with normalization of CK values and muscular weakness mostly recovered. Breast ultrasound and mammography showed a complete response to treatment and total body CT was negative for metastases. In April 2020 the patient underwent left mastectomy and axillary node resection with a pathologic (ypT0, ypN0, ycM0) and a radiologic complete response (shown in Figure 2), and full regression of dermatomyositis symptoms.

The patient is continuing her adjuvant therapy with trastuzumab administered subcutaneously (600 mg) every three weeks to complete one year of treatment and hormone therapy with aromatase inhibitor. Treatment with immunoglobulins is ongoing while prednisone has been reduced maintaining a complete clinical recovery and CK values within the limits of the norm (the last dosage was 98 IU/L).

## 2. Discussion

Dermatomyositis is an idiopathic inflammatory myopathy (IIM) characterized by subacute skin lesions, muscle weakness, and characteristic muscle biopsy findings. Gottron papules and heliotropic rash are typical features; other cutaneous manifestations are poikiloderma, holster sign, calcinosis cutis, psoriasiform changes and erythroderma [1,2]. More rare symptoms are edema, dysphagia and interstitial lung disease.

It has been observed that the presence of specific myositis-related autoantibodies (MSA) is associated with different clinical manifestations. The finding of high levels of anti-TF1γ and anti-NXP2 is frequent in paraneoplastic forms (about 80%) and often these patients have severe skin manifestations and dysphagia; a recent meta-analysis showed that patients with high levels of anti-TF1γ have a 27-fold higher odds ratio of developing malignancy [3,4].

From 15% to 30% of cases of dermatomyositis is a cancer-associated myositis (CAM) [5]. A new diagnosis of dermatomyositis in patients over 45 years of age should always be associated with oncological screening [6]. Dermatomyositis is most frequently associated with lung, ovarian, gastric, pancreatic, colorectal cancer and non-Hodgkin lymphoma; in women, 20% is associated with breast cancer [1,7]. The most represented histotype is ductal carcinoma while no statistical trends regarding hormone receptors status and HER2 positivity were detected [8]. Symptoms may precede the diagnosis of breast cancer, be simultaneous or appear later; reappearance of symptoms may indicate a relapse [9].

Management of early breast cancer (eBC) is primarily based on surgical approach that may be immediate or subsequent to neoadjuvant chemotherapy. The choice is based on the extent of disease and biological risk of recurrence. The chemosensitivity is variable according to phenotype (greater for HER2 positive tumors and poor for luminal-A like subtypes). Guidelines recommend evaluating neoadjuvant chemotherapy in all patients with HER2-positive disease >2 cm. Although neoadjuvant chemotherapy does not increase survival, it reduces the extent of surgery in locally advanced and large operable cancer and evaluates the therapeutic response, which is a well-established prognostic factor and guide the choice to adjuvant treatment [10].

Treatment of dermatomyositis is mainly based on corticosteroids and immunosuppressant agents and immunoglobulins as second-line treatment. In these patients, the treatment of cancer often shows the improvement of myositis [9,11].

Currently, there are no guidelines or randomized trials for the management of breast cancer complicated by dermatomyositis. The literature is based exclusively on case reports [1,2,4,6,8,11,12,13,14,15,16,17,18,19]. The existing reports do not clearly indicate whether an immediate or postponed surgical approach to neoadjuvant therapy is preferable. In our case the patient has done standard neoadjuvant chemotherapy for Stage III HER2 positive breast cancer. The choice was dictated by the extent of disease, the presence of mastitis, the lymph node involvement and HER2 positivity. The patient had rapid improvement of muscular symptoms after few chemotherapy administrations. There are no data about the role of neoadjuvant chemo/hormonal therapy and in previous case reports, surgery is preferred to neoadjuvant treatment, probably also due to the increased risk of infections [1,14]. However, the evidence for this is extremely low.

Moreover, in the case of patients with HER2+ disease, there is evidence to suggest an additional role of Trastuzumab in blocking the paraneoplastic process. Recently Pellegrino et al., published the case of a patient with HER2+ eBC and dermatomyositis who achieved the remission of symptoms only after the first administration of adjuvant Trastuzumab. The patient had high serum level of anti-HER2 antibodies. It could therefore be hypothesized that HER2-specific T-cell clones, stimulated by the presence of the HER2 antigen in the neoplasm, may cross-react with unknown skin antigens causing dermatomyositis. Probably trastuzumab has blocked this cross-reaction leading to dermatomyositis remission [19]. This supports our hypothesis that, not only is neoadjuvant chemotherapy safe in patients with paraneoplastic dermatomyositis, but that in the case of HER2 positivity trastuzumab could promote the remission of muscular symptoms.

## 3. Conclusions

This is an example of complete resolution of neurological symptoms and complete eradication of breast cancer after neoadjuvant chemotherapy and anti-HER2 therapy. These findings support the use of neoadjuvant chemotherapy when it is indicated, and also when associated with paraneoplastic dermatomyositis. Nevertheless, more data are warranted to establish the best management for these patients to gain much longer survival.

## Data Availability

No new data were created or analyzed in this study. Data sharing is not applicable to this article.

## References

[1] Dias L.P.N., Faria A.L.A., Scandiuzzi M.M., Inhaia C.L.d.S., Shida J.Y., Gebrim L.H. (2015). A rare case of severe myositis as paraneoplastic syndrome on breast cancer. World J. Surg. Oncol..

[2] Ahuja S., Makkar P., Gupta S., Vigoda I. (2016). Paraneoplastic syndrome and underlying breast cancer: A worsening rash despite initiation of chemotherapy. J. Community Support. Oncol..

[3] Trallero-Araguás E., Rodrigo-Pendás J.Á., Selva-O’Callaghan A., Martínez-Gõmez X., Bosch X., Labrador-Horrillo M., Grau-Junyent J.M., Vilardell-Tarrés M. (2012). Usefulness of anti-p155 autoantibody for diagnosing cancer-associated dermatomyositis: A systematic review and meta-analysis. Arthritis Rheum..

[4] Kubeček O., Soukup T., Paulík A., Kopecký J. (2016). Dermatomyositis with anti-TIF-1γ antibodies as a presenting symptom of underlying triple-negative breast cancer: A case report. BMC Cancer.

[5] Ikeda N., Yamaguchi Y., Kanaoka M., Ototake Y., Akita A., Watanabe T., Aihara M. (2020). Clinical significance of serum levels of anti-transcriptional intermediary factor 1-γ antibody in patients with dermatomyositis. J. Dermatol..

[6] Luu X., Leonard S., Joseph K.-A. (2015). Dermatomyositis presenting as a paraneoplastic syndrome with resolution of symptoms following surgical management of underlying breast malignancy. J. Surg. Case Rep..

[7] Hill C.L., Zhang Y., Sigurgeirsson B., Pukkala E., Mellemkjaer L., Airio A., Evans S.R., Felson D.T. (2001). Frequency of specific cancer types in dermatomyositis and polymyositis: A population-based study. Lancet.

[8] Hendren E., Vinik O., Faragalla H., Haq R. (2017). Breast cancer and dermatomyositis: A case study and literature review. Curr. Oncol..

[9] Osako T., Ito Y., Morimatsu A., Jinnin M., Tada K., Sakurai N., Takahashi S., Akiyama F., Sakamoto G., Iwase T. (2007). Flare-up of dermatomyositis along with recurrence of breast cancer. Breast J..

[10] Cardoso F., Kyriakides S., Ohno S., Penault-Llorca F., Poortmans P., Rubio I.T., Zackrisson S., Senkus E. (2019). Early breast cancer: ESMO Clinical Practice Guidelines for diagnosis, treatment and follow-up. Ann. Oncol..

[11] Lamquami S., Errarhay S., Mamouni N., Bouchikhi C., Banani A. (2015). Dermatomyositis revealing breast cancer: Report of a case. Pan Afr. Med. J..

[12] Khoo H.Y., Tan W.J., Cheong Y.T. (2018). Breast cancer with dermatomyositis as initial presentation. Med. J. Malays..

[13] Otsuka Y., Watanabe H., Kano Y., Tatebe N., Sunahori-Watanabe K., Kawabata T., Sada K.E., Wada J. (2017). Occurrence of dermatomyositis immediately after mastectomy subsequent to severe chemotherapeutic drug eruption. Intern. Med..

[14] Sandhu N.P., Zakaria S., Degnim A.C., Boughey J.C. (2011). Dermatomyositis presenting as a paraneoplastic syndrome due to underlying breast cancer. BMJ Case Rep..

[15] Song Y.J., Wu Y.F., Fan T. (2010). Dermatosis as the initial manifestation of malignant breast tumors: Retrospective analysis of 4 cases. Breast Care.

[16] Pectasides D., Koumpou M., Gaglia A., Pectasides M., Lambadiari V., Lianos E., Papaxoinis G., Economopoulos T. (2006). Dermatomyositis associated with breast cancer. Anticancer Res..

[17] Inaguma G., Shimada A., Tsunoda J., Matsuzaki T., Nishi T., Seki H., Matsumoto H. (2020). Inflammatory breast cancer associated with amyopathic dermatomyositis: A case report. Surg. Case Rep..

[18] Allouch A., Zaatarikahale T.B., Moussa M.K., Jounblat Y., Bitar N. (2020). Dermatomyositis: A Presenting Clinical Vignette in a Patient with Breast Cancer. Cureus.

[19] Pellegrino B., Mazzaschi G., Madeddu D., Mori C., Lagrasta C.A.M., Missale G., Quaini F., Musolino A. (2020). Clinico-Immunological Profile of a 67-Year-Old Woman Affected by HER2-Positive Breast Cancer and Autoimmune Dermatomyositis. Front. Oncol..

